# Demonstration of Spirochæta Pallida

**Published:** 1905-11

**Authors:** 


### DEMONSTRATION OF SPIROCHÆTA PALLIDA.

At the meeting of the Atlanta Society of Medicine October 5th, Drs. Taylor and Ballinger demonstrated under the microscope the micro-organism recently described by Schaudeim and Hoffmann, who claim it is a constant finding in the primary and secondary lesions of syphilis.

The lesions are washed and then a small amount of the scrapings are placed in a thin smear on a cover glass and fixed in alcohol twenty minutes. These are then stained for eighteen to twenty-four hours in azur I. and azur II. and Geimsa's Eosin.

The *Spirochœta Pallida* appears as a *very* thin corkscrew shape spirillum, faintly stained and is difficult to find—one specimen, however, showed nine in one field. In thickness they are smaller than the tubercle baccillus; in length they vary from the diameter of one to six red cells.

The slides that showed them most clearly were those in which the red corpuscles were stained a greenish slate color and the white corpuscles a deep purple.

They must be differentiated from the *spirochœta refringens*, which is larger, thicker and stains a distinct blue, while the *s. pallida* is a pinkish red, of high refractive character. Its curves are wider and less numerous. After once seeing the *spirochœta pallida* it can not be mistaken for the *spirochœta refringens*, or tissue shreds. Until once seen, however, certain tissue shreds cause considerable difficulty.

The specimens shown were from two chancres and one mucous patch. Eight slides showed the spirochæta. As to whether they are the cause of syphilis can not yet be positively stated, as they have not been isolated and cultivated and of course there must be a long series of cases by a number of independent workers before any conclusions can be drawn. The fact, however, that they have been found by reliable observers in many parts of Europe, and by Metchin Koff and Roux in the monkeys inoculated with syphilis and have not been found in any other lesions, seems to justify the view that they are perhaps the cause of syphilis.

---

ATLANTA is now the proud possessor of three regular medical schools, and those who in future shall languish on the bed of sickness will not suffer from a scarcity of doctors. This must be taken as a sign of prosperity and a growing recognition to Atlanta's claim to being the medical center of the South. It is to be hoped, however, that the presence of three schools of medicine in this city will not engender bitterness and factional rivalry nor by reason of competition deteriorate the standard of medical education and lower the dignity which the profession so merits and to which it aspires. The outlook for the medical profession in the United States was never more flattering than at present. It displays a growing solidarity which can only work for its upbuilding. The eyes of Europe are turning in our direction, and it is no longer necessary for the aspiring student to seek knowledge from the sources of science in the old country, for it can be obtained at home. These are things to think over in the multiplication of medical schools and the South has no desire to lag behind in the march of progress to higher and better things.

---

AT the annual meeting of the St. Francis Medical Association, held at Sherbrooke, Canada, during the week of September 23d, the following named were elected officers for the ensuing year: President, Dr. W. Larny; first vice-president, Dr. Ledoux; second vice-president, Dr. Edgar; secretary-treasurer, Dr. E. J. Williams; assistant secretary, Dr. Gadbout; council, Dr. Smith, Dr. Fregeau, and Dr. Pelletier.

